# Relationships Between Nutrition, Alcohol Use, and Liver Disease

**Published:** 2003

**Authors:** Charles S. Lieber

**Affiliations:** Charles S. Lieber, M.D., M.A.C.P., is a professor of medicine and pathology at Mount Sinai School of Medicine, and chief of the Section of Liver Disease and Nutrition as well as director of the Alcohol Research Center at the Bronx Veterans Affairs Medical Center, Bronx, New York

**Keywords:** alcoholic liver disorder, chronic AODE (alcohol and other drug effects), malnutrition, nutritional deficiency, MEOS (microsomal ethanol-oxidizing system), digestion, nutrient absorption, vitamins, vitamin therapy, liver function, ethanol metabolism, NAD, fatty liver, antioxidants, oxidative stress, S-adenosylmethionine

## Abstract

Many alcoholics are malnourished, either because they ingest too little of essential nutrients (e.g., carbohydrates, proteins, and vitamins) or because alcohol and its metabolism prevent the body from properly absorbing, digesting, and using those nutrients. As a result, alcoholics frequently experience deficiencies in proteins and vitamins, particularly vitamin A, which may contribute to liver disease and other serious alcohol-related disorders. Furthermore, alcohol breakdown in the liver, both by the enzyme alcohol dehydrogenase and by an enzyme system called the microsomal ethanol-oxidizing system (MEOS), generates toxic products such as acetaldehyde and highly reactive, and potentially damaging, oxygen-containing molecules. These products can interfere with the normal metabolism of other nutrients, particularly lipids, and contribute to liver cell damage. Nutritional approaches can help prevent or ameliorate alcoholic liver disease. For example, a complete balanced diet can compensate for general malnutrition. Administration of antioxidants (e.g., precursors of the endogenous antioxidant glutathione) can help the body eliminate reactive oxygen molecules and other reactive molecules generated from abnormal lipid breakdown. New agents currently are being studied as promising nutritional supplements for alcoholics with liver disease.

A complex interplay exists between a person’s alcohol consumption and nutritional status. Many people, including light-to-moderate drinkers who consume one to two glasses or less of an alcoholic beverage per day, consider those beverages a part of their normal diet and acquire a certain number of calories from them. When consumed in excess, however, alcohol can cause diseases by interfering with the nutritional status of the drinker. For example, alcohol can alter the intake, absorption into the body, and utilization of various nutrients. In addition, alcohol exerts some harmful effects through its breakdown (i.e., metabolism) and the resulting toxic compounds, particularly in the liver, where most of the alcohol metabolism occurs (Lieber 1992, 2000).

This article explores the relationships between a person’s alcohol consumption, nutritional status, and risk of alcoholic liver disease. It first describes the nutritional value of alcoholic beverages and discusses how alcohol consumption can contribute to malnutrition in heavy drinkers, with particular emphasis on the effects of alcohol on the digestion and absorption of various nutrients. The article then summarizes the general influence of a person’s nutrition on his or her liver function and explores the most important pathways of alcohol metabolism and their relationships with various nutritional factors. The article concludes by reviewing various current and emerging approaches in the nutritional management of alcoholic liver disease.

## The Nutritional Value of Alcoholic Beverages

Alcoholic beverages primarily consist of water, pure alcohol (chemically known as ethanol), and variable amounts of sugars (i.e., carbohydrates); their content of other nutrients (e.g., proteins, vitamins, or minerals) is usually negligible.^1^ Therefore, any calories provided by alcoholic beverages are derived from the carbohydrates and alcohol they contain. The carbohydrate content varies greatly among beverage types. For example, whiskey, cognac, and vodka contain no sugars; red and dry white wines contain 2 to 10 grams of sugar per liter (g/L); beer and dry sherry contain 30 g/L; and sweetened white and port wines contain as much as 120 g/L. Similarly, the alcohol content varies greatly among beverages, ranging from approximately 40 to 50 g/L in beer and coolers, to approximately 120 g/L in wine and prepacked cocktails, to 400 to 500 g/L in distilled spirits. An average drink— namely, 5 ounces (oz) of wine, 12 oz of beer, or 1.5 oz of distilled beverage— contains 12 to 14 grams of alcohol. Pure alcohol provides approximately 7.1 kilocalories per gram (kcal/g), compared with 4 kcal/g for carbohydrates. Thus, a 12-oz can of beer contains approximately 100 calories.

At least under certain conditions, however, alcohol-derived calories when consumed in substantial amounts can have less biologic value than carbohydrate-derived calories, as shown in a study in which Pirola and Lieber (1972) compared the weights of two groups of participants who received balanced diets containing equal numbers of calories. In one of the groups, 50 percent of total calories was derived from carbohydrates, whereas in the other group the calories were derived from alcohol.^2^ Although all participants received the same number of calories, those in the alcohol group exhibited a decline in body weight compared with those in the carbohydrate group. Moreover, when the participants received additional calories in the form of alcohol, they did not experience any corresponding weight gain. This suggests that some of the energy contained in alcohol is “lost” or “wasted”—that is, it is not available to the body for producing or maintaining body mass. Under other conditions, however, alcohol-derived calories have the same biologic value as calories derived from other nutrients. The various mechanisms involved and the circumstances in which alcohol calories fully count or do not count are described in detail elsewhere (Lieber 1991*a*).

Several mechanisms have been implicated in the apparent loss of alcohol-derived energy (Feinman and Lieber 1998). For example, some of the energy may be used up (wasted) during the breakdown of alcohol by a pathway known as the microsomal ethanol-oxidizing system (MEOS). (For more information on this system, see the section “Relationships Between Nutritional Factors and Alcohol Metabolism,” below.) As described later in this article, alcohol may damage the liver cells’ mitochondria—small membrane-enclosed cell structures that serve as the cell’s power plants—and these damaged mitochondria may waste energy during the breakdown of fats.

## The Nutritional Status of Alcoholics

**Figure 1:**
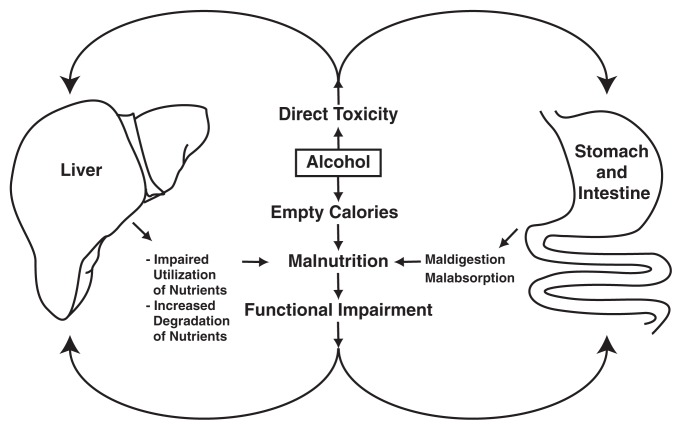
Interaction of alcohol’s direct toxic effects with malnutrition. In alcoholics, alcohol often replaces other nutrients (e.g., carbohydrates or proteins), resulting in insufficient intake of those nutrients (i.e., primary malnutrition), particularly because, under certain conditions, the calories provided by alcohol cannot be used effectively by the body—that is, they are “empty” calories. In addition, alcohol has direct toxic effects on the gastrointestinal tract and liver, leading to impaired digestion, reduced absorption of nutrients into the blood, and impaired utilization or increased degradation of those nutrients. These effects are referred to as secondary malnutrition and can contribute to the progression of liver damage. SOURCE: Lieber 1991*b*.

General observation suggests that many alcoholics do not consume a balanced diet; moreover, as mentioned earlier, excessive alcohol consumption may interfere with these alcoholics’ ability to absorb and use the nutrients they do consume. Accordingly, many alcoholics suffer from various degrees of both primary and secondary malnutrition. Primary malnutrition occurs when alcohol replaces other nutrients in the diet (described later in this section), resulting in overall reduced nutrient intake. Secondary malnutrition occurs when the drinker consumes adequate nutrients but alcohol interferes with the absorption of those nutrients from the intestine so they are not available to the body, as described in the following section (see figure 1).

The most severe malnutrition, which is accompanied by a significant reduction in muscle mass, generally is found in those alcoholics who are hospitalized for medical complications of alcoholism (e.g., alcohol-related liver disease or other organ damage). If these patients continue to drink, they will lose additional weight; conversely, if they abstain from drinking, they will gain weight. This pattern applies to patients with and without liver disease.

People who drink heavily but do not require hospitalization for alcohol-related medical problems, in contrast, often are not malnourished or show less severe malnutrition (Feinman and Lieber 1998). In these people, drinking, especially when accompanied by a high-fat diet and lack of physical activity, may actually lead to obesity of the trunk of the body. This relationship between heavy drinking and obesity has been observed particularly in women.

Overall, the wide range in nutritional status among alcoholics reflects, at least in part, the proportion of total calories they ingest in the form of alcohol as well as differences in what they eat. Moderate alcohol intake— that is, when alcohol accounts for up to 16 percent of total calories (i.e., approximately 320 kcal in a 2,000-kcal diet^3^)—is associated with slightly increased total energy intake. At this level of alcohol consumption, and even at slightly higher drinking levels (i.e., when alcohol accounts for up to 23 percent of total calories), the drinker typically substitutes alcohol for carbohydrates in the diet. In drinkers who consume more than 30 percent of their total calories in the form of alcohol, not only carbohydrate intake but also protein and fat intake decrease significantly. These drinkers’ consumption of vitamin A, vitamin C, and thiamine (vitamin B_1_) also may fall below the recommended daily allowances (Gruchow et al. 1985).

## Alcohol’s Effects on Digestion and Absorption of Essential Nutrients

Alcohol consumption, particularly at heavy drinking levels, not only influences the drinker’s diet but also affects the metabolism of those nutrients that are consumed. Thus, even if the drinker ingests sufficient proteins, fats, vitamins, and minerals, deficiencies may develop if those nutrients are not adequately absorbed from the gastrointestinal tract into the blood, are not broken down properly, and/or are not used effectively by the body’s cells. Two classes of nutrients for which such problems occur are proteins and vitamins.

### Amino Acids and Proteins

Proteins are essential components of all cells. They help maintain the cell’s structure, transport certain substances in and out of cells, and act as enzymes that mediate almost all biochemical reactions occurring in the cells. Proteins are composed of approximately 20 different building blocks called amino acids. Many of these amino acids can be produced by the body itself from various precursors or are recycled when proteins that are damaged or are no longer needed are broken down or degraded. Other amino acids (the so-called essential amino acids), however, must be acquired through diet. Alcohol can interfere with the uptake of these essential amino acids; indeed, studies using experimental animals have found that the animals absorbed less amino acid from the intestine after they received an alcohol dose (Adibi et al. 1992).

Patients with chronic liver failure (who in many cases are alcoholics) also exhibit a number of defects in protein metabolism. These include decreased production of proteins in the liver that are secreted into the blood (e.g., albumin and blood-clotting [i.e. coagulation] factors), decreased urea synthesis, and decreased metabolism of a group of amino acids called aromatic amino acids. These defects have important clinical consequences:

- Decreased production of the main protein found in the blood, albumin, may lead to abnormally low levels of this protein in the blood. Albumin is needed to help maintain normal blood volume as well as the blood’s concentrations of minerals and other dissolved molecules. Excessively low albumin levels may cause or exacerbate the abnormal accumulation of fluid in the abdomen (i.e., ascites) of patients with cirrhosis, which may worsen the impaired blood flow through the patient’s already damaged liver.
- Reduced levels of blood-clotting factors may predispose patients to the risk of internal bleeding in the gastrointestinal tract, which can have serious health consequences.
- Urea synthesis serves to remove from the body (by excreting it in the urine) the toxic ammonia that is generated during various metabolic reactions (including the breakdown of proteins). Reduced urea production, which results in excessive ammonia levels in the body, may increase the likelihood that patients develop altered brain function, a condition called hepatic encephalopathy. (For more information on hepatic encephalopathy, see the article by Roger Butterworth in this issue.)
- Abnormalities in the normal balance of various types of amino acids, such as increased levels of aromatic amino acids, also can increase the risk of hepatic encephalopathy.

Despite these abnormalities in protein metabolism, patients with cirrhosis do not require more protein from the diet than do people without cirrhosis (i.e., 35 to 50 g/day).

### Vitamins

Vitamins are molecules that are present in small amounts in various foods and are essential for normal metabolism; insufficient vitamin levels in the body can lead to serious diseases. Alcoholics, even without liver disease, tend to have clinical and/or laboratory signs of deficiencies in certain vitamins, particularly vitamins B_1_ (thiamine), B_2_ (riboflavin), B_6_ (pyridoxine), and C (ascorbic acid), as well as folic acid. The severity of these deficiencies correlates with the amount of alcohol consumed and with the corresponding decrease in vitamin intake. Vitamin deficiencies are especially common in patients with cirrhosis and result both from reduced intake with the diet and, at least for some vitamins, from reduced absorption of those vitamins that are ingested. One important example is the vitamin A deficiency frequently found in patients with cirrhosis.

Vitamin A (retinol), which is essential for bone growth and normal eye function, can be obtained directly from the diet or can be produced in the body from a precursor compound called beta-carotene.

#### Alcohol’s Effects on Vitamin A Levels

Numerous studies have assessed the effects of alcohol consumption on vitamin A and beta-carotene levels in the liver and blood. In the liver, both heavy alcohol consumption and use of other drugs can lead to reduced vitamin A levels. These drugs enhance the activity of the liver enzymes that break down vitamin A and similar molecules (Leo et al. 1987; Leo and Lieber 1999).

**Figure 2:**
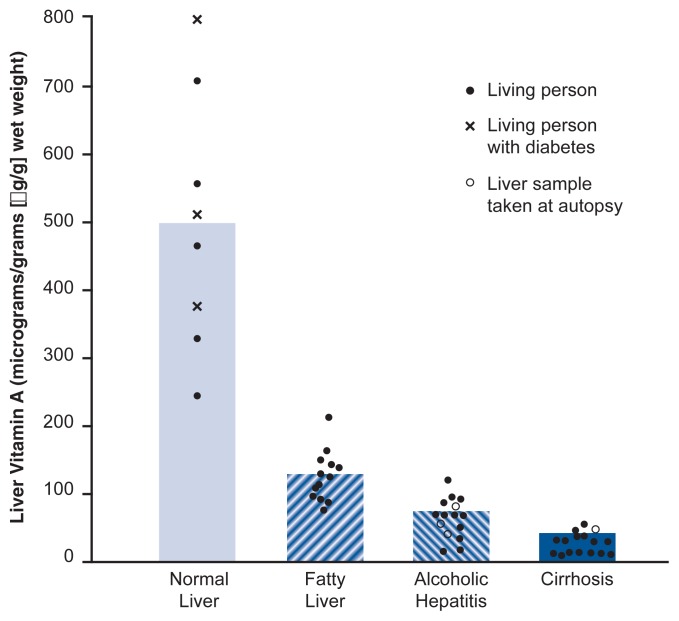
Vitamin A levels in the livers of healthy people and patients with various stages of alcoholic liver disease. The values were obtained by taking liver biopsies from study participants. Each circle or x represents one person; a filled circle represents a living participant, an open circle represents a sample taken during an autopsy of a deceased participant, and each x represents a study participant with a normal liver who had diabetes. The bars represent the mean value of all participants in each group. *P* values for the differences between the groups were all significant at the 0.001 level. SOURCE: Leo and Lieber 1982.

In the blood, short-term administration of alcohol results either in unchanged or increased vitamin A levels (Sato and Lieber 1981). Studies using baboons found that long-term feeding of alcohol raised the animals’ blood levels of beta-carotene (Leo et al. 1992), and increased beta-carotene levels in the blood also were found in human alcoholics (Ahmed et al. 1994). Other investigations compared the levels of beta-carotene, vitamin A, and other molecules related to these two compounds in the blood and livers of patients with alcoholic and nonalcoholic liver disease, normal livers of transplant donors, and blood from normal control subjects (Leo et al. 1993). The latter study found that the levels of vitamin A–related compounds, particularly vitamin A itself, were reduced in the livers of patients with liver disease (whether alcohol related or not) compared with the other groups (see figure 2). The decreases were greatest in patients with the most severe form of alcoholic liver disease (cirrhosis). Despite their reduced levels of vitamin A in the liver, however, many of these patients exhibited normal levels of beta-carotene in the blood, which suggests that liver disease alters the liver’s ability to take up beta-carotene and/or convert it into vitamin A. Impaired conversion of ingested beta-carotene to vitamin A in the liver during alcohol consumption may partially explain why the concentration of vitamin A in the liver is reduced, especially at advanced stages of alcoholic liver disease. In addition, alcohol promotes the secretion of vitamin A from the liver, thereby enhancing its decline in the liver (Leo et al. 1986).

As reviewed by Leo and Lieber (1999), studies of rats that were fed alcohol every day for several weeks confirmed that alcohol can reduce vitamin A levels in the liver. After receiving alcohol for 4 to 6 weeks, the animals’ vitamin A levels in the liver had declined by 60 percent. This reduction became even more severe (i.e., a 72-percent decline) after 7 to 9 weeks of alcohol administration. At the same time, the levels of vitamin A in the blood did not change. Even supplementing the animals’ diet with five times the usual amount of vitamin A could not prevent the alcohol-induced vitamin A depletion in the liver. Similar results were obtained in baboons that received 50 percent of their calories as alcohol. In these animals, vitamin A levels in the liver declined by 60 percent after 4 months and by 95 percent after 24 to 84 months.

#### Consequences of Altered Vitamin A Levels

Vitamin A deficiency can impair the ability of the eye to adjust to dark conditions (i.e., causing night blindness) and can result in other eye disorders. In the liver, reduced vitamin A levels can change the structures of components of some cells, and these changes may be exacerbated by the consumption of alcohol (Leo et al. 1983). However, excess vitamin A also has harmful effects. For example, in the liver, increased vitamin A levels can promote the formation of scar tissue (i.e., fibrosis) (Leo and Lieber 1983), which also is worsened by concurrent alcohol use (Leo et al. 1982).

Alcohol has varying effects on vitamin A and beta-carotene content and metabolism throughout the body. For example, alcohol increases the vitamin A content of some tissues and decreases vitamin A in other tissues. In addition, alcohol can speed up or alter the conversion of vitamin A to other compounds. Some or all of these changes may contribute to alcohol’s toxic effects on the liver and to the development of liver fibrosis.^4^

#### Vitamin A Therapy

Because alcohol consumption leads to reduced vitamin A levels in the liver, with potentially detrimental effects, it would appear plausible to treat alcoholics with extra vitamin A to compensate for alcohol’s effects. However, several factors complicate vitamin A therapy in the setting of alcoholism:

- It is difficult to assess how much vitamin A actually is stored in the tissues, because vitamin A in the blood does not necessarily reflect levels in the liver.
- High doses of vitamin A are toxic.
- Even usual doses of vitamin A are potentially harmful in alcoholics who continue to drink, because alcohol potentiates the toxicity of vitamin A.

Therefore, only modest doses of vitamin A should be given to patients who may continue to drink or use other drugs. Patients with night blindness who have low levels of vitamin A in the blood may be given 2 mg of vitamin A per day for several weeks as a possible therapy. Treatment with zinc also may be necessary, especially in patients with night blindness, because this mineral is needed for vitamin A metabolism.

To avoid or reduce the problems associated with vitamin A therapy, clinicians also have considered treating alcoholics with the vitamin A precursor beta-carotene. However, although beta-carotene is thought to be less hazardous, it also can cause toxic effects in the livers of patients who continue to use alcohol (Leo et al. 1992). In addition, beta-carotene increases the risk of lung cancer in smokers (Alpha-Tocopherol Beta-Carotene Cancer Prevention Study Group 1994). This is important because most smokers also drink alcohol, and researchers have found that the increased lung cancer risk after beta-carotene therapy was related to the smokers’ concurrent alcohol use (Albanes et al. 1996). For this reason, beta-carotene therapy of these patients must be used cautiously.

## A Person’s Nutrition Affects Liver Function

Malnutrition, regardless of its causes, can lead to liver damage and impaired liver function. For example, children in underdeveloped countries whose diets do not contain enough protein can develop a disease called kwashiorkor. One symptom of this disorder is the accumulation of fat in the liver, a condition known as fatty liver. Studies performed during and after World War II indicated that severe malnutrition also could lead to liver injury in adults. However, in these cases other factors, including exposure to certain toxins or parasites that are prevalent in war-ravaged or underdeveloped countries, may have exacerbated the relationship between liver injury and poor nutrition.

Because malnutrition also is common in alcoholics, clinicians initially thought that malnutrition, rather than alcohol itself, was responsible for alcohol-induced liver injury. Over the past 40 years, however, a more balanced view has evolved. Studies in humans, primates, and rodents have established that alcohol can cause liver damage even in well-nourished people (Lieber 1992). Moreover, controlled studies using hospitalized participants have demonstrated that even subjects receiving an enriched diet could develop fatty liver if the carbohydrates in the diet were replaced with alcohol. Finally, epidemiological analyses have found a close correlation between per capita alcohol consumption and the likelihood of cirrhosis, indicating that alcohol itself contributes to liver disease.

It is becoming clear that nutritional effects and the toxic effects of alcohol often are intertwined at the biochemical level. For example, alcohol induces the MEOS to break down alcohol, but this breakdown also leads to the previously mentioned waste of energy observed in alcoholics who replace carbohydrates in their diet with alcohol. Similarly, alcohol promotes the breakdown of nutrients such as vitamin A, of which alcoholics may already consume too little with their diet.

## Relationships Between Nutritional Factors and Alcohol Metabolism

**Figure 3:**
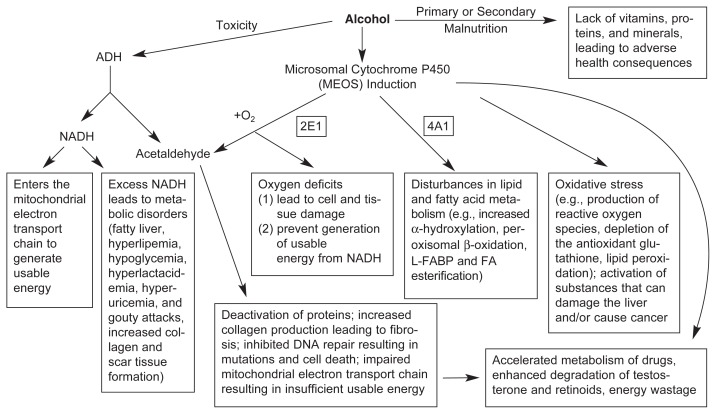
Effects of heavy alcohol consumption caused by alcohol-related malnutrition and alcohol breakdown by the enzyme alcohol dehydrogenase (ADH) and the microsomal ethanol-oxidizing system (MEOS). Alcohol consumption can lead to primary and secondary malnutrition as described in figure 1. Alcohol breakdown by ADH results in the formation of excess levels of the molecule reduced nicotinamide adenine dinucleotide (NADH), which can cause various metabolic problems. Moreover, both ADH and the MEOS convert alcohol to acetaldehyde, a toxic molecule that has numerous adverse effects. Alcohol also enhances the activity of the central enzyme of the MEOS, cytochrome P450 2E1, which exacerbates some of the toxic effects of acetaldehyde and generates a harmful condition called oxidative stress in the cells. Oxidative stress is characterized by excess levels of reactive oxygen species (ROS), abnormal lipid breakdown resulting in additional reactive molecules, and/or reduced levels of antioxidants (e.g., glutathione) which can eliminate reactive molecules. SOURCE: Modified from Lieber 1998.

As indicated in the previous sections, complex interactions exist between alcohol and its metabolism and other nutritional and metabolic factors. In the liver, alcohol is broken down primarily through two pathways: the enzyme alcohol dehydrogenase (ADH) and the MEOS (for more information on these two pathways, see the sidebar “Pathways of Alcohol Metabolism”). Both of these pathways have several nutritional and metabolic consequences in heavy drinkers (see figure 3).

**Pathways of Alcohol Metabolism**

Alcohol is broken down (i.e., metabolized) in the liver primarily through two pathways: the alcohol dehydrogenase (ADH) pathway and the microsomal ethanol-oxidizing system (MEOS). In people who consume alcohol at moderate levels and/or only occasionally, most of the alcohol is broken down by ADH, an enzyme found in the fluid that fills the cell (i.e., the cytosol). ADH converts alcohol (chemically known as ethanol) to acetaldehyde, a toxic and highly reactive molecule. During this reaction, hydrogen is removed from the alcohol and transferred to a molecule called nicotinamide adenine dinucleotide (NAD), converting it to reduced NAD (NADH). As described in the main article, NADH participates in numerous other metabolic reactions, passing on the hydrogen to other compounds, and excess cellular NADH levels have harmful effects on those cells. Subsequently, the acetaldehyde is converted to acetate by a second enzyme, aldehyde dehydrogenase.

The MEOS plays a role in alcohol metabolism, particularly after higher alcohol consumption. As the name implies, the reactions that make up the MEOS occur in microsomes, small sphere-shaped vesicles that are split off from a membrane-enclosed cell structure called the endoplasmic reticulum, which serves to transport molecules through and out of the cells. The main component of the MEOS is the enzyme cytochrome P450, which, like ADH, converts alcohol to acetaldehyde. This reaction also relies on oxygen and a molecule called reduced nicotinamide adenine dinucleotide phosphate (NADPH) and results in the formation of NADP and water. As byproducts of these reactions, highly reactive, oxygen-containing molecules called oxygen radicals or reactive oxygen species (ROS) are generated. These ROS can contribute to liver damage through a variety of mechanisms.

Although the rate at which ADH breaks down alcohol generally stays the same, the activity of the MEOS can be increased (i.e., induced) by alcohol consumption. Because the MEOS metabolizes not only alcohol but also other compounds (e.g., certain medications), enhanced MEOS activity resulting from high alcohol consumption also can alter the metabolism of those medications. This may contribute to harmful interactions between alcohol and those medications or otherwise influence the activity of those medications.

Of the several variants of cytochrome P450, a form called CYP2E1 is most prominent in alcohol metabolism. The activity of this molecule can increase up to fourfold following alcohol consumption (Tsutsumi et al. 1989). Other types of cytochrome P450, such as CYP1A2 and CYP3A4, also are involved in the breakdown of alcohol (Salmela et al. 1998).

—Charles S. Lieber

ReferencesSalmelaKSKessovaIGTsyrlovIBLieberCSRespective roles of human cytochrome P–4502E1, 1A2 and 3A4 in the hepatic microsomal ethanol oxidizing systemAlcoholism: Clinical and Experimental Research222125213219989884161TsutsumiMLaskerJMShimizuMThe intralobular distribution of ethanol inducible P450IIE1 in rat and human liverHepatology104374461989267396910.1002/hep.1840100407

### The ADH Pathway

The ADH pathway, which converts alcohol to the toxic substance acetaldehyde in a reaction that releases hydrogen atoms, is responsible for most of the alcohol breakdown in liver cells. However, how fast alcohol is broken down by this pathway depends, at least in part, on nutritional factors. For example, low-protein diets reduce the levels of ADH in the liver, lowering the rate of alcohol breakdown both in humans and in laboratory animals (Bode et al. 1971). Prolonged fasting also has been shown to decrease the rate of alcohol breakdown in isolated rat liver cells. These observations suggest that for any given alcohol dose, malnourished alcoholics break down the alcohol more slowly and therefore develop higher blood alcohol levels, and sustain them longer, than well-nourished subjects. Because the effects of alcohol on the body depend on blood alcohol levels, reduced alcohol degradation may lead to more severe damage to the liver and other organs.

Conversely, alcohol metabolism by the ADH pathway also may influence metabolic functions. As mentioned above, ADH-mediated breakdown of alcohol generates hydrogen atoms in addition to acetaldehyde. These hydrogen atoms interact with a molecule called nicotinamide adenine dinucleotide (NAD), converting it to reduced NAD (NADH). NADH, in turn, participates in many essential biochemical reactions in the cell, and in the process passes on its hydrogen to other molecules. For proper functioning of the cell, the ratio of NAD to NADH must be tightly controlled. When alcohol metabolism generates excess amounts of NADH, the cell can no longer maintain the normal NAD/NADH ratio. This altered NAD/NADH ratio may lead to several metabolic disorders (see figure 3) (Lieber 1992). For example, elevated levels of NADH cause the formation of abnormally high levels of lactic acid, which in turn reduce the capacity of the kidney to excrete uric acid. Excessive uric acid in the body can exacerbate gout, a disorder characterized by extremely painful swelling of certain joints. Therefore, alcohol-induced increases in NADH levels and, subsequently, uric acid levels, which can be worsened by other alcohol-induced metabolic effects, may at least partly explain the common clinical observation that excessive alcohol consumption causes or aggravates attacks of gout.

In addition, increased NADH promotes the generation of the building blocks of fat molecules (i.e., fatty acids) and reduces the breakdown of fats in the liver, thereby contributing to fat accumulation in that organ (Lieber and Schmid 1961). Other alcohol-related mechanisms also contribute to fat accumulation in the liver, including:

- Decreased excretion of fat-containing proteins from the liver
- Release of fats from other tissues, which then are transported to the liver
- Enhancement of the liver’s uptake of fats circulating in the blood.

The resulting fatty liver is the earliest stage and the most common form of alcohol-induced liver disease.

In addition to contributing to the development of fatty liver, the increases in NADH levels resulting from the ADH-mediated breakdown of alcohol also may play a role in the formation of scar tissue that characterizes fibrosis, a more severe stage of liver disease. This relationship was suggested by the observation that a molecule that can capture hydrogen away from NADH completely prevents certain liver cells (i.e., stellate cells) from producing elevated levels of molecules that contribute to the formation of scar tissue (Casini et al. 1991).

### The Microsomal Ethanol-Oxidizing System (MEOS)

After moderate alcohol consumption, most of the ingested alcohol is broken down by the ADH pathway described above. After chronic heavy alcohol consumption, the MEOS pathway of alcohol metabolism becomes more important. This pathway consists of several enzymes located in the liver microsomes—small spherical structures found in all cells. The MEOS has been investigated extensively because its activity increases substantially after long-term alcohol consumption and because it is important for the breakdown and elimination of other foreign molecules from the body, including certain medications (for a review, see Lieber 1997). Therefore, activation of the MEOS after alcohol consumption may alter the breakdown of those medications and may contribute to harmful interactions between alcohol and those medications.

The primary component of the MEOS is the molecule cytochrome P450, which exists in several variants. The variant most important for alcohol metabolism is cytochrome P450 2E1 (CYP2E1). Studies using liver biopsies from people who recently had been drinking alcohol found that the levels of CYP2E1 were four times higher in these subjects than in control subjects who had not been drinking alcohol (Tsutsumi et al. 1989). In contrast, the levels of ADH in the liver did not change following alcohol consumption.

Enhanced CYP2E1 activity in response to chronic alcohol consumption (or other factors) probably contributes to the development of alcoholic liver disease. Alcoholics commonly suffer from a type of liver disease called steatohepatitis, which is an inflammation of the liver with concurrent fat accumulation in the liver. Steatohepatitis also is frequently found in people with diabetes and excessive or morbid obesity, even if they are not alcoholics. Studies have found that, in addition to breaking down alcohol, CYP2E1 also mediates certain steps in the metabolism of fatty acids as well as of chemicals called ketones (e.g., acetone) and that acetone, like alcohol, can stimulate CYP2E1 activity (Koop and Casazza 1985). Patients with diabetes or morbid obesity commonly have higher than normal levels of fatty acids and ketones. This observation suggests that, in nonalcoholics, steatohepatitis can be the end result of enhanced CYP2E1 activity caused by excess levels of ketones and fatty acids; in alcoholics, steatohepatitis can result from enhanced CYP2E1 activity caused by chronic heavy drinking.

Alcohol-induced activation of the MEOS also contributes to alcoholic liver disease through other mechanisms. For example, alcohol breakdown by CYP2E1 generates several types of highly reactive oxygen-containing molecules called reactive oxygen species (ROS) (see figure 3). These ROS can damage liver cells by inactivating essential enzymes and altering the breakdown of fat molecules; higher ROS levels contribute to a condition called oxidative stress, which can cause liver cell damage. These ROS effects are exacerbated if the body’s normal defense systems against this damage— antioxidants, such as glutathione (GSH) and vitamin E (α-tocopherol)— also are impaired. Alcohol and its metabolism have been shown to reduce the levels of both GSH and vitamin E. For example, the breakdown product of alcohol, acetaldehyde, lowers GSH levels in the liver. Furthermore, patients with cirrhosis have reduced amounts of vitamin E in the liver (Leo et al. 1993). Thus, alcohol metabolism through the MEOS can lead to liver damage both by generating harmful substances (e.g., the ROS) and by reducing the levels of protective substances (e.g., GSH).

## Nutritional Management of Alcoholic Liver Disease

As discussed in the previous sections, alcohol consumption and alcohol metabolism can lead to harmful effects on the liver through numerous pathways related to the drinker’s nutrition and metabolism. Alcoholic liver disease typically develops in several sequential and partially overlapping stages. The first stage, fatty liver, is characterized by fat accumulation in the liver; it is sometimes associated with inflammation, and is called steatohepatitis or alcoholic hepatitis, when severe. At this stage, liver cells may begin to die and scar tissue may form, leading to the next stage of liver disease, fibrosis. Excessive scar tissue formation, in turn, eventually destroys the normal liver structure, resulting in cirrhosis, the most severe type of liver disease.

Treatment of alcoholic liver disease must be started as early as possible in the disease process because patients are more likely to die as the disorder advances. For example, one study of patients with alcoholic liver disease found that 70 percent of patients with fatty liver still were alive after 4 years, whereas less than 50 percent of patients with cirrhosis still were alive after the same amount of time (Chedid et al. 1991). If the cirrhosis was associated with inflammation (i.e., alcoholic hepatitis), the outlook was even worse, with only about 33 percent of patients still alive after 4 years. Unfortunately, these high mortality rates, higher than those for many cancers, attract relatively little attention from the public or the medical profession because many people believe that no effective treatment of alcoholic liver disease is available. However, new insights into the mechanisms contributing to the disorder have resulted in prospects for improved treatments, including nutritional management approaches that can lead to better outcomes.

### Management of Nutritional Deficiencies

Many drinkers who consume more than 30 percent of their total calories as alcohol ingest less than the recommended daily amounts of carbohydrates; proteins; fats; vitamins A, C, and B (especially thiamine); and minerals, such as calcium and iron. Deficiencies in these essential nutrients may exacerbate the effects of alcohol itself, resulting in serious disorders. To prevent these deficiencies, clinicians can provide alcoholics with a complete diet comparable to that of nonalcoholics. Even a complete, balanced diet, however, cannot prevent some of the organ damage that results from alcohol’s direct toxic effects, including alcoholic liver disease.

Nevertheless, dietary supplements may prevent or ameliorate some of alcohol’s harmful effects. For example, brain damage resulting from a lack of vitamin B_1_ (thiamine), which can lead to conditions such as Wernicke-Korsakoff syndrome, can be reversed to some extent. Vitamin B_1_ generally can be administered with a great margin of safety; therefore, all alcoholics undergoing treatment should be presumed to have a vitamin B_1_ deficiency and should receive 50 mg of thiamine per day (either by injection if the patients are hospitalized or by mouth). Alcoholics also should receive supplements of vitamins B_2_ (riboflavin) and B_6_ (pyridoxine) at the dosages usually found in standard multivitamin preparations. Adequate folic acid levels can in most cases be achieved with a normal diet, unless there is evidence of a severe deficiency. As discussed before, vitamin A should be given only to those alcoholics who have a well-documented deficiency and who can stop or at least moderate their alcohol consumption, because of the potential harmful effects of vitamin A when combined with alcohol.

In addition to an improved diet to reverse nutritional deficiencies, alcoholics with moderate malnutrition also might benefit from treatment with anabolic steroids (Mendenhall et al. 1995). These compounds, which are derived from the male hormone testosterone, can be used in the short term to promote overall body buildup and therefore may help the alcoholic recover from malnutrition.

### Prevention of Fatty Liver

As mentioned, alcohol’s interference with the normal metabolism of fatty acids promotes the deposit of dietary fat in the liver. Consequently, decreasing the amount of fat in the diet can reduce the severity of the alcoholic fatty liver (Lieber and DeCarli 1970). Another means of influencing the extent of alcoholic fatty liver is to change the type of fats consumed. For example, researchers found that consumption of fat molecules known as long-chain triglycerides promotes fatty liver, whereas medium-chain triglycerides (MCTs) significantly reduce alcoholic fatty liver. This difference probably results from the fact that MCTs are more likely to be broken down in the body than long-chain triglycerides and therefore are less likely to be deposited in the liver (Lieber et al. 1967). Animal studies have confirmed that MCTs can protect against fat deposition in the liver (Nanji et al. 1996). Thus, providing a diet rich in MCTs may be a promising therapeutic approach, particularly for relatively short-term interventions in patients who are recovering from alcohol-induced liver injury. MCTs generally are available only in health food stores as a dietary supplement.

### Antioxidant Therapy to Reduce Oxidative Stress

Alcohol-induced oxidative stress in the liver cells plays a major role in the development of alcoholic liver disease. This condition results from several processes related to alcohol metabolism:

- Changes in the NAD/NADH ratio resulting from alcohol breakdown by ADH.
- Production of ROS during alcohol breakdown by the MEOS. This is particularly important after chronic alcohol consumption, which stimulates the activity of the MEOS.
- Reduced levels of the antioxidant GSH in the liver. GSH is a small molecule consisting of three amino acids, including cysteine. Acetaldehyde, the first product of alcohol breakdown, can bind to GSH and specifically to cysteine, thereby removing active GSH from the liver cells (Shaw et al. 1983). In addition, alcohol itself inhibits the production of new GSH.

**Figure 4:**
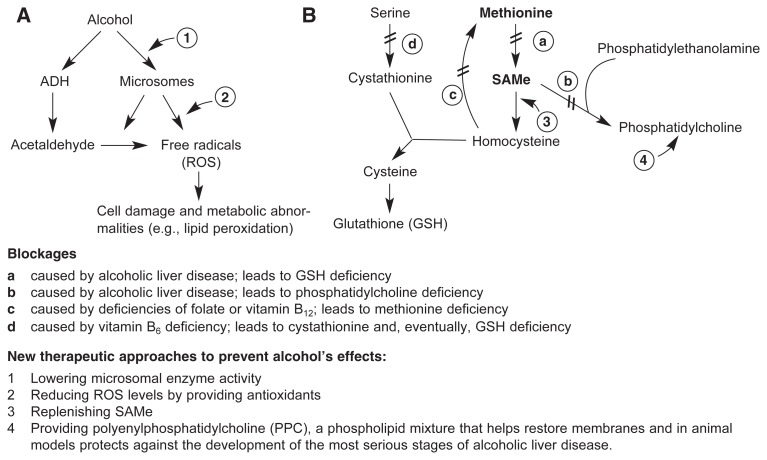
Alcohol’s effects on the levels of reactive molecules and the antioxidant glutathione (GSH) in the cell. **(A)** Alcohol breakdown by the enzyme alcohol dehydrogenase (ADH) and by the microsomal ethanol-oxidizing system (MEOS) generates acetaldehyde, a reactive molecule that among other harmful effects interacts with cysteine, preventing it from being used to generate GSH (see panel B). Both the MEOS and acetaldehyde also lead to the generation of reactive oxygen species (ROS) that damage the cells through various mechanisms (e.g., lipid peroxidation). ROS can be eliminated or converted to harmless substances by GSH and other antioxidants. **(B)** One of the precursors of GSH is the amino acid methionine, which first is converted to S-adenosylmethionine (SAMe). SAMe then is further modified to yield cysteine. Alcohol consumption and alcoholic liver disease cause the blocks labeled a and b; both folate and vitamin B_12_ deficiency cause block c; vitamin B_6_ deficiency causes block d; and all these blockages interfere with GSH production. Administration of SAMe can help raise GSH levels in the cells.

Both increased ROS production and GSH depletion lead, among other harmful effects, to the abnormal breakdown of fat molecules (i.e., lipid peroxidation). This process results in the formation of toxic compounds that can stimulate scarring and damage liver cells, thereby contributing to alcoholic liver disease. Accordingly, it is important to prevent or reduce the oxidative stress associated with alcohol metabolism. One approach to achieve this is to ensure that the cells have adequate levels of antioxidants, particularly GSH, that can “capture” ROS and break them down or convert them to less harmful molecules. Because GSH depletion plays a key role in alcoholic liver injury, it is therapeutically important to increase GSH levels in the liver. GSH cannot be administered directly, however, because the molecule cannot penetrate directly into the liver cells. Similarly, the amino acid cysteine, which is most important for ensuring adequate GSH levels, cannot be used as a supplement because it cannot enter the liver cells. Therefore, clinicians have tried to administer precursors of cysteine (see figure 4), such as the compound acetylcysteine or the molecule S-adenosylmethionine (SAMe) (discussed in the following section), which can reach the cells and be converted to cysteine there.

Another important antioxidant is vitamin E. Alcoholics with cirrhosis often have low vitamin E levels in the liver (Leo et al. 1993), whereas alcoholics without cirrhosis generally have vitamin E levels within the normal range. Therefore, administration of vitamin E supplements may be useful only for some alcoholics. Moreover, studies in baboons have found that animals with normal vitamin E levels in the liver still developed fibrosis or even cirrhosis (Lieber et al. 1994). Vitamin E also showed no positive effect in a trial of patients with alcoholic cirrhosis who received supplements of the compound (de la Maza et al. 1995). These observations suggest that although vitamin E deficiency increases the liver’s vulnerability to alcohol, normal vitamin E levels may not be able to prevent the developmenst of alcoholic liver disease, particularly fibrosis.

### Emerging Therapies

#### S-adenosylmethionine (SAMe)

Because nutritional supplementation of the antioxidant GSH or its component cysteine is not an effective way to ensure adequate GSH levels in the livers of alcoholics, investigators have looked for other compounds that can promote GSH production. The ultimate precursor of cysteine is the amino acid methionine (figure 4). Before cysteine is generated, methionine is converted in the cell to SAMe; however, the enzyme that mediates this reaction is much less active in patients with liver disease (Martin-Duce et al. 1988). Consequently, administration of methionine itself is not useful in these patients; in fact, excess methionine can have some adverse effects on liver function.

Because patients with alcoholic liver disease can produce little SAMe, and the existing SAMe is used up rapidly to generate new GSH, SAMe deficiency typically develops in the cells of these patients. This deficiency can be corrected, however, by administering supplemental SAMe (Lieber 2002). The effectiveness of this approach has been shown in both animals and humans. In baboons, SAMe administration resulted in a corresponding improvement in alcohol-induced liver injury, as shown by less GSH depletion as well as by changes in the activities of certain enzymes that serve as indicators of liver function, and by the production of fewer abnormal mitochondria (Lieber et al. 1990). In humans, a clinical trial in which SAMe was given to patients with alcoholic cirrhosis according to strict scientific standards also achieved significant therapeutic success (Mato et al. 1999). When patients with the most severe liver disease were excluded, those who received SAMe were significantly less likely to die or require a liver transplant within the next 2 years than were patients who had received an inactive substance (i.e., a placebo). Moreover, the study detected virtually no harmful side effects of SAMe treatment. Therefore, this approach appears to hold promise for the treatment of patients with alcoholic liver disease and should be investigated further.

#### PPC

One of the harmful consequences of alcohol breakdown by the MEOS is the formation of ROS, which among other effects can cause lipid peroxidation. Not all fat molecules, however, are equally sensitive to peroxidation. For example, polyunsaturated fats are more susceptible than monounsaturated or saturated fats. With fat molecules containing additional phosphate groups (phospholipids), however, the opposite may occur—polyunsaturated phospholipids may be particularly resistant to peroxidation. This hypothesis is supported by studies evaluating the effects of the compound polyenylphosphatidylcholine (PPC) in animal models. In these studies, PPC, which is a mixture of molecules known as phosphatidylcholines (extracted from soybeans), prevented lipid peroxidation (Aleynik et al. 1997) and attenuated the associated liver injury in rats that had been treated with hepatic toxins (Ma et al. 1996). Furthermore, PPC decreased oxidative stress (Lieber et al. 1997) and prevented the development of alcohol-induced cirrhosis in baboons (Lieber et al. 1994). Clinical trials currently are being conducted to test the effectiveness of PPC in the treatment of alcoholic liver disease.

#### Silymarin

Another antioxidant that has shown positive results in experimental animals (Lieber et al. 2003) is a molecule called silymarin, the active constituent of milk thistle. Some clinical trials have shown that this compound has beneficial effects such as improved survival in patients with alcoholic liver disease (Ferenci et al. 1989). Other controlled studies, however, have not verified such an action (Pares et al. 1998). Additional clinical trials to determine the usefulness of this compound for treating alcoholics with liver disease now are under way.

## Summary

Chronic drinkers, particularly those who consume a substantial portion of their daily calories in the form of alcohol, often show evidence of malnutrition such as deficits in amino acids, proteins, and certain vitamins. These deficits can derive from an inadequate diet as well as from alcohol’s effects on these nutrients and their metabolism. Particularly common is a deficit in vitamin A, which is required for proper eye function and bone growth. Moreover, both vitamin A deficits and excessive vitamin A levels can lead to liver damage, including fibrosis. Therefore, administering vitamin A to correct a deficiency is difficult and should be controlled carefully, particularly in the presence of alcohol abuse, which exacerbates vitamin A’s toxicity.

Potentially harmful compounds, which, in combination with other nutritional factors, can lead to liver damage and other alcohol-related disorders, are generated not only by alcohol itself but also by its metabolism via ADH or the MEOS. Particularly important is the MEOS, which, among other functions, influences fat metabolism. Chronic alcohol consumption activates the MEOS and may thereby contribute to the development of a fatty liver. Other byproducts of MEOS-mediated alcohol degradation, such as ROS, also alter fat metabolism and damage the liver by promoting lipid peroxidation.

Because alcoholics frequently have poor nutritional status, which is further exacerbated by alcohol’s effects on the body’s metabolism, nutritional approaches may be useful in the treatment of alcoholic patients, including those with alcoholic liver disease. Possible approaches include nutritional supplementation to compensate for deficits in nutrients, as well as administration of antioxidants to counteract the alcohol-induced increase in oxidative stress and the resulting liver damage. Because of the potential usefulness of such an approach, several new compounds currently are being studied in clinical trials. If they prove effective, these nutritional management approaches could be important tools in the prevention or amelioration of alcoholic liver disease.

## Footnotes

1 Because they provide almost no nutrients, alcoholic beverages are considered “empty calories.”

2 The study participants were observed on the metabolic ward of a hospital during the experiments. The quantity of alcohol administered did not exceed the amount routinely consumed by these volunteers.

3 Dietary guidelines published by the U.S. Department of Agriculture and the Department of Health and Human Services define moderate drinking as consumption of no more than one standard drink per day by women and no more than two standard drinks per day by men. Depending on the specific calorie content of the drinks consumed, the definition of moderate drinking used here (i.e., up to 16 percent of total calories) may exceed the amounts specified in the dietary guidelines.

4 Alcohol-mediated alterations of vitamin A status also may play a role in the association of low vitamin A or beta-carotene levels with certain types of cancer (Lieber 1987).
